# Impacts of vessel-generated waste on seasonal dynamics of water quality in the Kirtonkhola River, Bangladesh

**DOI:** 10.1515/biol-2025-1380

**Published:** 2026-10-01

**Authors:** Eti Khan Mitu, Md Nayem Hasan Munna, Sufiya Khatun, Sk. Shahnewaz Zimi, Hafiz Ashraful Haque, Rattan Lal

**Affiliations:** Coastal Studies and Disaster Management, University of Barishal, Barishal, 8254, Bangladesh; CFAES Rattan Lal Center for Carbon Management and Sequestration (Lal Carbon Center), School of Environment and Natural Resources, College of Food, Agricultural, and Environmental Sciences, The Ohio State University, Columbus, OH, USA; Soil and Environmental Sciences, University of Barishal, Barishal, 8254, Bangladesh; Department of Water Resources Development, Institute of Water and Flood Management (IWFM), Bangladesh University of Engineering and Technology (BUET), Dhaka, 1000, Bangladesh

**Keywords:** water quality, vessel waste, seasonal variation, Kirtonkhola River, physicochemical parameters, pollution assessment

## Abstract

Rivers play a vital role in sustaining ecosystems and human livelihoods, yet increasing anthropogenic activities threaten their ecological integrity. This study assessed seasonal variations and relationships among key water quality parameters, including pH, electrical conductivity (EC), total dissolved solids (TDS), and dissolved oxygen (DO) in the Kirtonkhola River, Bangladesh, to evaluate seasonal water-quality patterns in relation to passenger vessel activities and associated waste discharge. Monthly samples were collected from two sites with contrasting vessel activity between March 2018 and February 2019. Results showed clear seasonal patterns, with pH ranging from 5.92 to 7.69, EC from 165.33 to 357 μS/cm, TDS from 80 to 183.33 mg/L, and DO from 4.23 to 7.46 mg/L. Lower pH and DO during parts of the monsoon and higher EC and TDS during the dry season reflected seasonal physicochemical variability potentially influenced by hydrological conditions and anthropogenic inputs. Linear regression confirmed a strong relationship between EC and TDS (*R* ^2^ = 0.983 and 0.975, *p* < 0.001). All parameters remained within standard limits, highlighting the need for continued monitoring and improved vessel waste management.

## 1 Introduction

Rivers serve as vital natural resources supporting human livelihoods, biodiversity, transportation, agriculture, aquaculture, and economic activities. However, anthropogenic influences have increasingly degraded river water quality, making it a critical global environmental concern [1]. Rivers in densely populated regions are particularly vulnerable to pollution because of increasing urbanization, transportation activities, and human dependence on river systems [2]. Rivers also provide important ecological functions, including sediment transport, ecotourism, sustaining aquatic habitats, and supporting fisheries and recreational activities [3], 4]. Nevertheless, increasing human activities and overexploitation of water resources continue to threaten water quality and ecosystem health [5].

The direct and indirect disposal of pollutants from industrial, agricultural, and domestic sources has significantly altered the physicochemical and biological properties of surface waters [6]. Rivers are frequently used as waste disposal sites, which can negatively affect aquatic ecosystems and water usability [7]. Poor waste management practices, particularly in developing regions lacking strict environmental regulation, further intensify river pollution problems [8]. In many developing countries, including Bangladesh, vessel-generated waste is an emerging yet underexplored source of river pollution. Passenger vessels generate sewage, graywater from kitchens, sinks, showers, and laundry, and food wastes, which may introduce organic matter, nutrients, dissolved constituents, detergents, and other contaminants into receiving waters. Such discharges can affect physicochemical water-quality characteristics, including pH, electrical conductivity (EC), total dissolved solids (TDS), and dissolved oxygen (DO), particularly through organic loading and changes in dissolved constituents [9]. The Kirtonkhola River, a major waterway in Barishal, experiences substantial passenger vessel activity and associated anthropogenic pressures. Elevated EC and TDS values may reflect increased ionic concentrations from vessel-related discharges, whereas reduced DO levels may indicate oxygen depletion caused by microbial decomposition of organic matter [10]. Although petroleum-derived contaminants and heavy metals may also occur in vessel-dominated environments, the present study primarily focused on physicochemical indicators associated with organic and ionic pollution because these parameters directly reflect changes in river water chemistry and ecological condition [3].

Despite growing concerns regarding river pollution in Bangladesh, limited quantitative information is available on the seasonal dynamics and physicochemical signatures associated with vessel-related waste discharge. Most previous studies have focused primarily on industrial pollution, salinity intrusion, or heavy metal contamination, whereas comparatively little attention has been given to transport-related organic and ionic pollution in river systems influenced by intense passenger vessel activity. In particular, there is insufficient understanding of how vessel-associated discharges influence key water quality parameters such as pH, EC, TDS, and DO under contrasting seasonal hydrological conditions.

Seasonal hydrological variability can substantially influence river water quality and modify the effects of anthropogenic inputs. During the monsoon, increased freshwater inflow can dilute dissolved constituents, resulting in lower EC and TDS concentrations [11]. In contrast, reduced freshwater inflow and increased evaporation during the dry season can increase mineralization and dissolved ion concentrations [12]. Similar hydrological influences on salinity and water chemistry have been reported in other aquatic systems [13]. These seasonal processes highlight the importance of distinguishing broader hydrological variability from localized anthropogenic influences when evaluating river water quality. Accordingly, integrated water-quality assessment and management approaches have been applied internationally [14] and in Bangladesh [15] to better understand and manage anthropogenic pressures on aquatic systems.

The objective of this study was to evaluate seasonal variations in physicochemical water quality parameters of the Kirtonkhola River in relation to passenger vessel activities and associated waste discharge. The study primarily focused on organic and ionic pollution originating from domestic and sanitary vessel waste, which may influence EC, TDS, pH, and DO dynamics. It was hypothesized that areas with higher vessel activity would exhibit increased EC and TDS concentrations and lower DO levels due to elevated organic loading and dissolved ion enrichment. Strong positive correlations among EC, TDS, and pH were expected to indicate ionic enrichment from vessel waste discharges, whereas weak or negative correlations with DO would reflect oxygen depletion associated with organic loading. It was further hypothesized that the analyzed heavy metal concentrations would remain below instrumental detection limits, indicating limited measurable dissolved heavy-metal contamination during the study period.

## 2 Materials and methods

### 2.1 Study area and sampling sites

This study was conducted in the Kirtonkhola River, Barishal, Bangladesh (Figure 1), an essential waterway supporting transportation, commerce, and local livelihoods. Two distinct sampling sites were selected based on their exposure to direct waste disposal from passenger vessels. The first site, Launch Ghat (22.69933°N, 90.37605°E), is a high-traffic docking area where passenger vessels frequently dispose of waste directly into the river, making it more susceptible to pollution.

**Figure 1:**
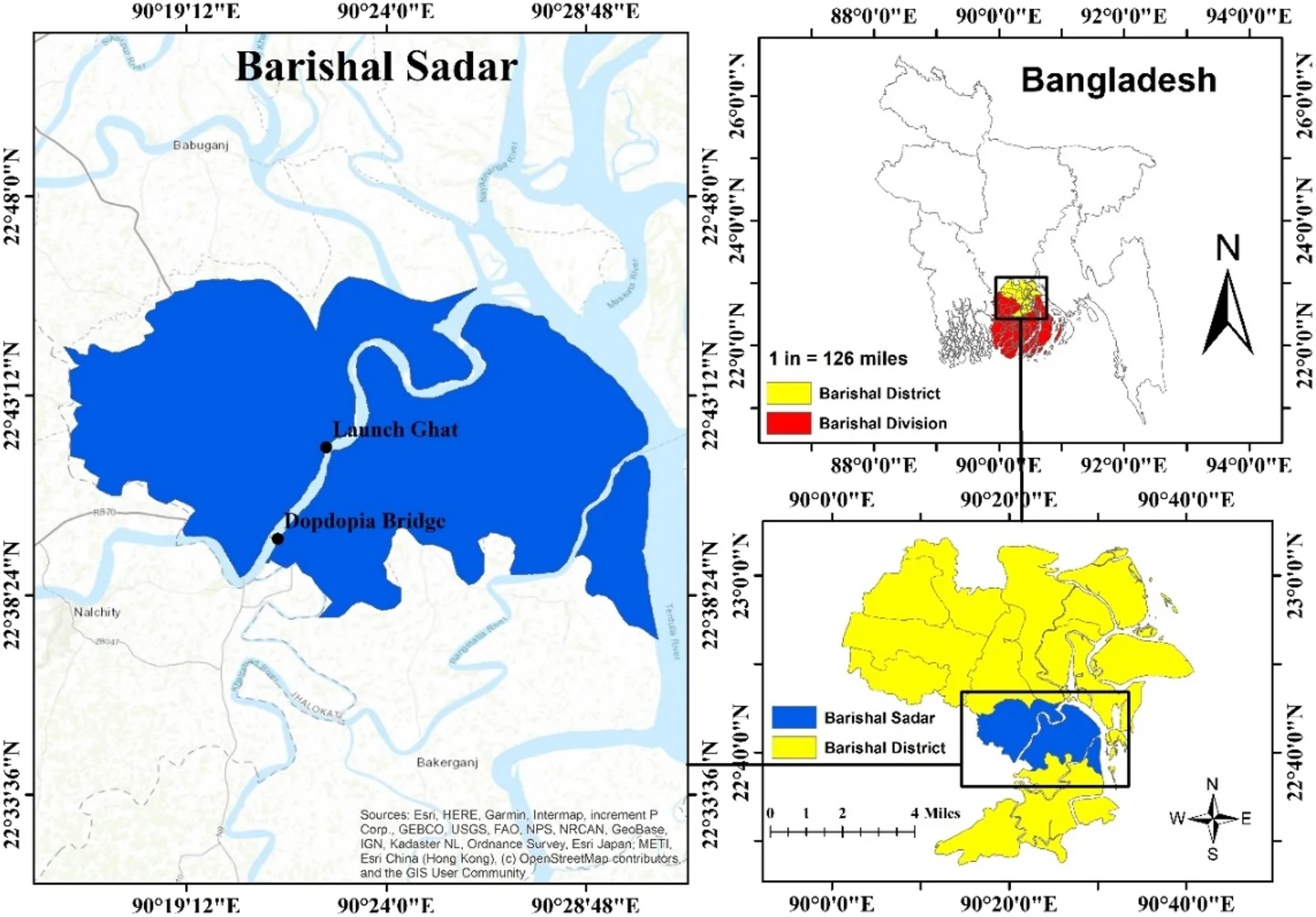
Map of the study area showing the sampling locations, Launch Ghat and Dopdopia Bridge, in the Kirtonkhola River, Barishal Sadar, Bangladesh.

The second site, Dopdopia Bridge (22.66272°N, 90.35663°E), is located comparatively farther from the main passenger launch terminal and was observed to experience comparatively lower visible vessel congestion and docking activity during the sampling period. Therefore, it was considered a comparatively less impacted observational site for relative comparison rather than an absolute pollution-free reference location. These geographical coordinates offer essential reference points for navigation and spatial analysis in the region.

Monthly water samples were collected from both sites over a one-year period (March 2018 – February 2019) to assess seasonal variations in water quality. The geographic locations of both sites were recorded using Global Positioning System (GPS) coordinates to ensure sampling consistency throughout the study. Basic hydrological parameters such as water depth, flow velocity, and turbulence were not measured during the sampling campaign because of equipment limitations. Therefore, seasonal hydrological processes, including monsoon-driven dilution, flow variability, and turbulence-related reaeration, were considered qualitatively during interpretation of the physicochemical results.

### 2.2 Sample collection and preservation

Water samples were collected from the surface layer (0–20 cm depth) using pre-cleaned polyethylene bottles (500 mL capacity). To minimize contamination, each bottle was thoroughly rinsed three times with river water before sample collection. After collection, samples were stored in an icebox at 4 °C and transported to the laboratory for further analysis. To maintain the integrity of DO measurements, the bottles were filled completely without air bubbles and fixed immediately using Winkler reagents to prevent changes during transport. The sampling design focused only on surface water conditions and did not include vertical profiling or bottom-water sampling because of logistical and equipment limitations. Therefore, potential vertical variability in physicochemical properties and sediment-water interactions could not be evaluated in the present study.

### 2.3 Water quality analysis

The study analyzed key physicochemical parameters, including pH, EC, TDS, and DO, to evaluate the water quality of the Kirtonkhola River. All measurements were performed in triplicates to ensure analytical precision and reliability.

pH was measured using a calibrated digital pH meter (Hanna HI 98107) with standard buffer solutions of pH 4.0, 7.0, and 10.0 prior to each measurement. EC was determined using a portable EC meter (Hanna HI 8033), and readings were expressed in microsiemens per centimeter (µS/cm). TDS and DO were measured using a calibrated digital multimeter (HACH 51910), with results expressed in milligrams per liter (mg/L). Water temperature was not measured during the study period because of instrument limitations. Therefore, the potential influence of temperature on DO solubility and EC variability could not be quantitatively evaluated and should be considered when interpreting seasonal physicochemical trends.

In addition to the physicochemical parameters, concentrations of selected heavy metals (Fe, Pb, Zn, As, Co, and Hg) were determined following standard laboratory procedures using Inductively Coupled Plasma–Optical Emission Spectroscopy (ICP-OES) following USEPA Method 200.7. The instrument was calibrated using multi-element standards, and method detection limits ranged between 0.0001 mg/L and 0.005 mg/L depending on the element analyzed. All measurements were performed in triplicate to ensure analytical precision. However, the concentrations of all analyzed metals remained below the instrumental detection limits throughout the study period at both sampling sites.

### 2.4 Data analysis

Statistical analyses were conducted to evaluate seasonal and site-specific variations in water quality parameters. Descriptive statistics, including mean and standard deviation, were computed to summarize the dataset. One-way analysis of variance (ANOVA) was applied to determine significant differences among sampling months, with a significance threshold of *p* < 0.05 for all tests. Although the statistical approach effectively captured seasonal trends and parameter relationships, more advanced analyses incorporating both spatial and temporal variability could further improve understanding of river pollution dynamics in future investigations. Pearson’s correlation analysis was performed to examine interrelationships among key water quality parameters (pH, EC, TDS, and DO) at both Launch Ghat and Dopdopia Bridge. In addition, a simple linear regression analysis was used to quantify the relationship between EC and TDS, with coefficients of determination (*R* ^2^) and *p*-values used to evaluate the strength and significance of the association. Model assumptions, including normality and homoscedasticity, were verified through residual diagnostics to ensure statistical validity. All statistical analyses were performed using IBM SPSS Statistics 25, while graphical visualizations, such as trend analysis and seasonal variations, were prepared using SigmaPlot 15.0.

## 3 Results and discussion

### 3.1 pH

pH is a critical parameter influencing the metabolic activities of aquatic organisms, as it directly affects enzymatic reactions, physiological functions, and overall ecosystem stability [16]. In aquatic environments, pH serves as a key indicator of water quality and pollution levels in watershed areas. According to the Department of Environment [17], the recommended pH range for inland surface water is 6.5–8.5, while the acceptable limits for irrigation, fish culture, and recreational activities vary between 6.0 and 9.5 [18], 19]. Water bodies with pH values outside these thresholds may experience ecological imbalances, affecting aquatic biodiversity and water usability [20].

In this study, the mean pH values at both sampling sites remained within the standard range, although variations were observed across different months (Table 1). At Station A (Barishal Launch Ghat), pH values ranged from 5.92 ± 0.07 (June) to 7.69 ± 0.02 (March), while at Station B (Dopdopia Bridge), the highest pH was recorded in March (7.62 ± 0.09) and the lowest in June (5.90 ± 0.08). A significant decline in pH was observed between March and June, reaching its lowest point in June, followed by a sharp increase from June (5.92 ± 0.07 and 5.90 ± 0.08) to July (6.56 ± 0.15 and 6.66 ± 0.04). By September, pH levels at both stations stabilized at 6.97 ± 0.03 (Figure 2A). The steep decline in pH during the monsoon months is likely attributed to excessive rainfall, which introduces acidic components through atmospheric deposition and dilution effects, lowering the buffering capacity of the river [21], 22]. Rainwater contains dissolved minerals and atmospheric pollutants that interact with river water, forming acidic compounds that contribute to pH reduction [23].

**Table 1:** Monthly variations in pH, EC, DO, and TDS at Launch Ghat and Dopdopia Bridge from March 2018 to February 2019. Error bars represent standard deviation (SD), indicating the natural variability of water quality parameters across sampling months.

Months	pH		EC (µS/cm)		DO (mg/L)		TDS (mg/L)	
	Launch Ghat	Dopdopia Bridge	Launch Ghat	Dopdopia Bridge	Launch Ghat	Dopdopia Bridge	Launch Ghat	Dopdopia Bridge
Mar 18	7.69 ± 0.02^a^	7.62 ± 0.09^a^	261.00 ± 7.81^bc^	237 ± 38.22^bcd^	5.24 ± 0.45^cde^	4.25 ± 0.77^c^	136.67 ± 5.77^bc^	120.00 ± 17.32^bc^
Apr 18	7.54 ± 0.03^ab^	7.45 ± 0.17^ab^	308.67 ± 0.58^a^	357 ± 8.72^a^	5.24 ± 0.35^cde^	5.86 ± 1.16^abc^	160.00 ± 0.00^a^	183.33 ± 5.77^a^
May 18	6.22 ± 0.26^g^	6.49 ± 0.7^d^	177.00 ± 1.73^fg^	169.33 ± 1.53^cd^	4.41 ± 0.29^e^	4.23 ± 0.25^c^	90.00 ± 0.00^g^	90.00 ± 0.00^cd^
June 18	5.92 ± 0.07^g^	5.9 ± 0.08^cd^	165.33 ± 0.58^g^	165.67 ± 0.58^d^	4.92 ± 1.86^de^	5.28 ± 1.94^bc^	80.00 ± 0.00^h^	80.00 ± 0.00^d^
July 18	6.56 ± 0.15^f^	6.66 ± 0.44^bcd^	180.00 ± 3.61^fg^	172.67 ± 7.02^cd^	7.30 ± 0.16^ab^	7.36 ± 0.23^ab^	90.00 ± 0.00^g^	86.67 ± 5.77^cd^
Aug 18	6.60 ± 0.09^ef^	6.77 ± 0.08^abcd^	201.33 ± 31.90^ef^	222.33 ± 6.11^bcd^	7.39 ± 0.13^a^	7.46 ± 0.07^a^	106.67 ± 5.77^f^	113.33 ± 5.77^bcd^
Sep 18	6.97 ± 0.03^cd^	6.97 ± 0.03^abc^	221.33 ± 1.53^de^	225.67 ± 1.53^bcd^	7.46 ± 0.03^a^	7.45 ± 0.14^a^	110.00 ± 0.00^f^	116.67 ± 5.77^bcd^
Oct 18	6.92 ± 0.06^cde^	7.04 ± 0.05^abc^	244.67 ± 7.57^cd^	232 ± 13.75^bcd^	7.29 ± 0.23^ab^	7.17 ± 0.07^ab^	126.67 ± 5.77^de^	116.67 ± 5.77^bcd^
Nov 18	6.90 ± 0.17^def^	6.86 ± 0.21^abc^	239.33 ± 1.53^cd^	241.67 ± 1.53^bc^	7.23 ± 0.51^ab^	7.29 ± 0.18^ab^	120.00 ± 0.00^e^	120.00 ± 0.00^bc^
Dec 18	7.14 ± 0.10^cd^	7.09 ± 0.04^abc^	253.33 ± 0.58^bc^	248.67 ± 1.53^b^	6.75 ± 0.05^abc^	7.00 ± 0.07^ab^	130.00 ± 0.00^cd^	130.00 ± 0.00^b^
Jan 19	7.08 ± 0.05^cd^	7.33 ± 0.11^abc^	275.67 ± 1.15^b^	269.67 ± 6.43^b^	6.56 ± 0.50^abcd^	6.70 ± 0.45^ab^	140.00 ± 0.00^b^	140.00 ± 0.00^b^
Feb 19	7.26 ± 0.13^bc^	7.16 ± 0.57^abc^	319.33 ± 11.93^a^	259.67 ± 75.06^b^	5.51 ± 0.24^bcde^	6.11 ± 0.27^abc^	160.00 ± 0.00^a^	133.33 ± 37.86^b^

Environmental quality standards, pH (6.5–8.5, DoE, 1997), EC (7,000 μS/cm, DoE, 1997; 8,000–10,000 μS/cm, ADB, 1994), and DO (4.0–6.0 mg/L, De 2005). Irrigation water quality standards; pH (6–8.5), EC (0–3,000 μS/cm), and TDS (2,000 mg/L, ADB, 1994). Different lowercase superscript letters indicate significant differences among monthly means within each parameter and sampling location (*p* < 0.05); means sharing at least one letter are not significantly different.

**Figure 2:**
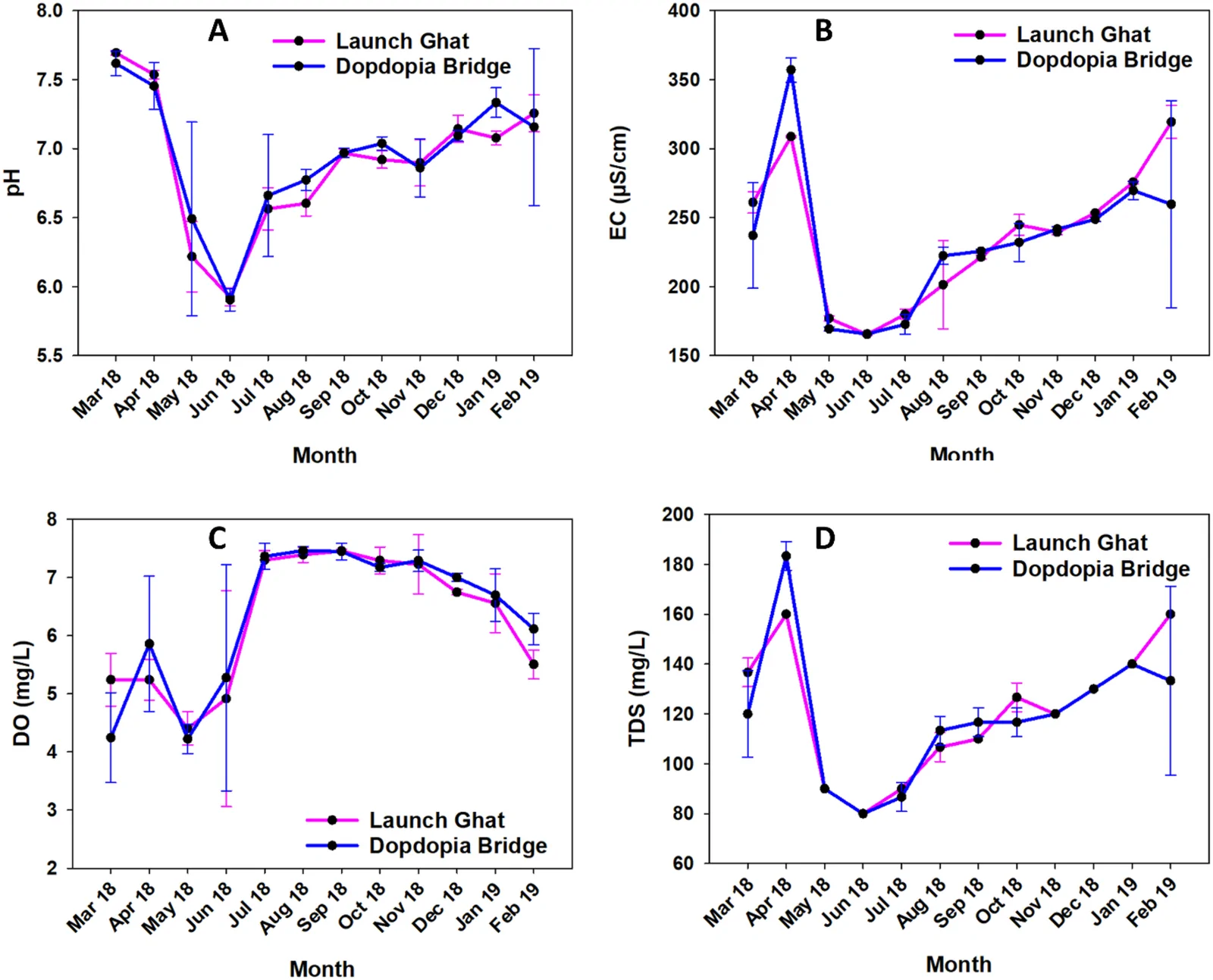
Seasonal variations in pH (A), EC (B), DO (C), and TDS (D) at Launch Ghat and Dopdopia Bridge (March 2018 – February 2019). Mean values with SD error bars show pH and DO decline during the monsoon, while EC and TDS rise in the dry season, reflecting hydrological influences.

Fluctuations in pH significantly influence chemical and biological processes in aquatic environments. Deviations from the optimal pH range can alter the solubility and bioavailability of heavy metals such as copper (Cu), lead (Pb), and arsenic (As), as well as toxic compounds like ammonia (NH_3_^+^) [24], 25]. These changes can lead to increased toxicity, affecting aquatic flora and fauna by disrupting metabolic pathways, impairing reproduction, and increasing mortality rates [26]. Additionally, pH fluctuations influence nutrient cycling and microbial activity, potentially altering primary production and trophic interactions in river ecosystems [27].

The observed gradual increase in pH from July to September suggests a recovery phase following the monsoon, likely driven by reduced rainfall, increased carbonate buffering, and decreased acid input from runoff [28]. From August to February, the average pH remained within acceptable limits, indicating a relatively stable water chemistry during the dry season. However, the lower pH values observed during June–July may indicate seasonal physicochemical conditions that could influence aquatic ecosystem functioning, particularly under prolonged acidic conditions associated with increased rainfall and runoff during the monsoon period [29], 30].

Although pH values generally remained within acceptable environmental limits, noticeable seasonal fluctuations were observed at both Launch Ghat and Dopdopia Bridge. The relatively small spatial differences between the two sites indicate that broader hydrological and seasonal processes likely played a greater role in regulating pH dynamics than localized vessel activity alone. Nevertheless, the observed variations still suggest a partial influence of vessel-related and anthropogenic inputs on the river’s physicochemical condition. Periods of comparatively lower pH during certain months emphasize the importance of continuous monitoring to prevent potential acidification risks and protect aquatic ecosystem health. Variability in pH may result from a combination of point-source pollutants, including industrial effluents and vessel waste discharge, as well as non-point sources such as agricultural runoff and acid precipitation. Therefore, integrated watershed management strategies, including regulation of acidic discharges, establishment of riparian buffer zones, and improved waste management practices, are essential for maintaining ecological stability and preserving the water quality of the Kirtonkhola River for both biodiversity conservation and human use [31].

### 3.2 EC

EC is a crucial parameter in assessing water quality as it indicates the concentration of dissolved ions, minerals, and chemical compounds [32]. The observed EC values in this study exhibited seasonal fluctuations across the two sampling sites. The highest EC value was recorded in April (357 ± 8.72 μS/cm) at Dopdopia Bridge, while at Launch Ghat, the peak EC value was 319.33 ± 11.93 μS/cm in February. The average EC levels for the entire study period were 237.25 μS/cm at Launch Ghat and 233.44 μS/cm at Dopdopia Bridge, indicating that EC values were generally comparable between the two sampling sites, although Launch Ghat exhibited slightly higher values during several sampling months (Figure 2B). The seasonal variation of EC revealed a sharp decline from April to May at both sites, followed by a gradual increase from June to February at Dopdopia Bridge. In contrast, Launch Ghat displayed periodic fluctuations in EC, indicating potential local environmental influences.

The variability in EC across different months is primarily influenced by hydrological and climatic conditions, including precipitation, upstream water inflow, and anthropogenic activities [33]. The declining EC from April to May is likely due to early monsoon rainfall, which increases surface runoff and dilutes the ionic concentration in the river. Conversely, the gradual rise in EC from June to February suggests progressive mineralization and lower freshwater inflow during the dry season [32].

When compared to standard EC guidelines, the measured values were significantly lower than the Bangladesh Department of Environment [17] standard of 1,200 μS/cm for inland surface water. Additionally, EC levels were well below the thresholds set for recreational activities (500 μS/cm), irrigation (750 μS/cm), and aquaculture (800–1,000 μS/cm) [18]. These findings indicate that the river water is not excessively mineralized and may be suitable for general ecological functioning. However, very low EC levels can signal poor nutrient availability, which may affect aquatic biodiversity [33].

The low EC values observed at both sites suggest minimal ionic contamination, which is consistent with the hydrological origin of the river system [32]. Many rivers in Bangladesh originate from Indian tributaries, and during the dry season, limited freshwater inflow reduces the transport of dissolved minerals into the study sites. This explains why EC levels remained moderate to low throughout the study period. While low EC levels do not pose a direct risk to human health, they indicate a low concentration of dissolved salts and essential minerals, which could influence aquatic productivity [17]. Certain fish species and microorganisms require moderate ionic strength for survival, and extremely low EC levels may limit their distribution in these waters [18].

Furthermore, the suitability of this water for agricultural use is questionable. Low EC levels suggest low mineral content, which may necessitate the use of fertilizers to enhance soil productivity [33]. However, excessive fertilizer application can lead to nutrient runoff, contaminating the river during the monsoon season. Anthropogenic activities such as agricultural runoff and wastewater discharge can alter EC levels over time, warranting continuous monitoring [32]. Overall, the findings indicate that EC at both Launch Ghat and Dopdopia Bridge followed a seasonal pattern strongly influenced by hydrological variability, particularly rainfall dilution and dry-season mineral concentration. Although vessel-related discharges may have contributed to localized ionic enrichment, the relatively small differences between sites suggest that hydrological controls were also important drivers of EC variability. These results partially supported the hypothesis regarding increased ionic concentrations under higher vessel activity conditions.

### 3.3 DO

DO is an important indicator of aquatic ecosystem condition, with low concentrations potentially causing physiological stress to aquatic organisms. DO levels varied significantly across seasons and locations. At Launch Ghat, the highest DO value was recorded in September (7.46 ± 0.03 mg/L), whereas at Dopdopia Bridge, the peak DO value (7.46 ± 0.07 mg/L) occurred in August. A gradual decline in DO was observed from September to February at both stations, indicating seasonal changes in oxygen availability. The lowest DO levels were recorded in May, with values of 4.41 ± 0.29 mg/L at Launch Ghat and 4.23 ± 0.25 mg/L at Dopdopia Bridge (Figure 2C). A sharp drop in DO from April to May was particularly evident at Dopdopia Bridge, while Launch Ghat experienced a more gradual decrease in DO levels over the same period.

DO is a fundamental water quality parameter that reflects the physical, chemical, and biological processes occurring in an aquatic system [34]. It plays a crucial role in sustaining aquatic life, as most fish and aquatic organisms require free oxygen (O_2_) for respiration. According to APHA [35], an average DO level of 6.5 mg/L generally indicates good water quality, while DO concentrations below 1 mg/L are unsuitable for fish survival. The standard DO range for fish culture is 5–6 mg/L, with values below 4 mg/L leading to stress or mortality in sensitive aquatic species [19], 36].

The observed variations in DO levels can be attributed to seasonal changes, biological activity, and organic matter decomposition in the river system. Photosynthesis, respiration, and organic decomposition play key roles in determining DO levels [37]. During the monsoon and post-monsoon seasons (August–September), increased photosynthetic activity by aquatic plants and phytoplankton enhances oxygen production, leading to peak DO concentrations [35]. The high DO levels recorded during this period suggest favorable water quality conditions for aquatic life.

Conversely, the lowest DO values were observed in May, coinciding with the onset of summer. Seasonal warming can accelerate microbial decomposition and increase oxygen consumption associated with organic matter degradation [38], 39]. Reduced river flow and turbulence can also limit atmospheric reaeration and contribute to lower DO concentrations [17]. Therefore, the lower DO values observed in May may reflect the combined influence of seasonal warming, organic matter decomposition, and hydrological conditions. However, because water temperature, flow velocity, and turbulence were not directly measured in this study, their individual contributions to the observed DO variation could not be quantitatively determined.

Comparing the results with standard DO requirements, the measured values were mostly within the recommended range for fish and livestock (4.0–6.0 mg/L) [17]. However, the low DO values in May indicate a stressful condition for aquatic organisms, particularly species sensitive to oxygen depletion. According to Meade [19], fish experience physiological stress when DO levels drop below 4.0 mg/L, making certain periods of the year less suitable for fish survival and aquaculture activities.

### 3.4 TDS

TDS represent the concentration of dissolved inorganic and organic substances in water, including carbonates, bicarbonates, chlorides, phosphates, and nitrates of calcium (Ca), magnesium (Mg), sodium (Na), potassium (K), and manganese (Mn), along with organic matter, salts, and suspended particles [40]. The standard TDS thresholds for different water uses are 1,000 mg/L for drinking water, 1,500 mg/L for industrial use, 5,000 mg/L for livestock, and 2,000 mg/L for irrigation [18]. The observed TDS values fluctuated seasonally across the two sampling locations. At Launch Ghat, the highest TDS concentration (160 ± 0.00 mg/L) was recorded in February, while the lowest (80 ± 0.00 mg/L) occurred in June. Similarly, at Dopdopia Bridge, the maximum TDS value (183.33 ± 5.77 mg/L) was recorded in April, whereas the lowest (80 ± 0.00 mg/L) was observed in June. The mean TDS concentrations at Launch Ghat and Dopdopia Bridge were 120.84 mg/L and 119.17 mg/L, respectively, indicating relatively similar dissolved solid content at both sites (Figure 2D). The seasonal pattern showed that TDS levels increased from June to February and declined sharply between April and May at both locations. This seasonal variation suggests a dynamic influence of hydrological processes, rainfall, and dilution effects on dissolved solids in the river.

The variation in TDS levels is primarily influenced by runoff, precipitation, mineral dissolution, and biological activity. Higher TDS values typically indicate increased concentrations of dissolved bicarbonates, sulfates, and chlorides of Ca, Mg, and Na [41], whereas lower values suggest dilution effects and reduced mineralization. The low TDS values recorded in June at both stations coincide with the onset of the monsoon season, where increased rainfall leads to higher dilution and reduced ion concentrations in the river [40].

The gradual increase in TDS from June to February aligns with progressive evaporation, reduced rainfall, and increased mineralization during the dry season. In contrast, the sharp decline between April and May suggests a sudden influx of freshwater, potentially from seasonal rainfall, which diluted the dissolved solids concentration. These findings are consistent with the EC trends, further confirming that mineral concentrations remain low throughout the study period.

When compared to standard TDS guidelines, the recorded values at both sites were well below the recommended levels for drinking water (1,000 mg/L), irrigation (2,000 mg/L), and industrial use (1,500 mg/L) [18]. This indicates that mineral concentrations in the river are significantly lower than required for agricultural or industrial applications.

The implications of low TDS levels extend to aquatic life and water usability. For aquaculture purposes, extremely low TDS may be unsuitable for fish growth, as some species require a minimum ionic concentration for metabolic functions [41]. Additionally, low TDS suggests a deficiency in essential nutrients like calcium, magnesium, and bicarbonates, which are necessary for aquatic ecosystems. Overall, the TDS results generally supported the observed seasonal ionic variability in the river system; however, the relatively modest differences between sites suggest that seasonal hydrological processes likely exerted stronger influence on dissolved solid concentrations than localized pollution sources alone.

### 3.5 Heavy metal concentrations in water

The concentrations of trace and potentially toxic metals, including iron (Fe), lead (Pb), zinc (Zn), arsenic (As), cobalt (Co), and mercury (Hg), were monitored monthly at both Launch Ghat and Dopdopia Bridge from March 2018 to February 2019 (Table 2). All analyzed metals were below the detection limits of the analytical instruments (Fe: 0.01 mg/L, Pb: 0.001 mg/L, Zn: 0.005 mg/L, As: 0.001 mg/L, Co: 0.002 mg/L, Hg: 0.0001 mg/L) throughout the study period, indicating that all analyzed metals remained below the instrumental detection limits throughout the study period.

**Table 2:** Summary of trace and potentially toxic metals in the Kirtonkhola River (March 2018 – February 2019).

Metal	Symbol	Detection limit (mg/L)	Launch Ghat	Dopodopia Bridge
Iron	Fe	0.001–0.005	BDL	BDL
Lead	Pb	0.0005–0.002	BDL	BDL
Zinc	Zn	0.0003–0.002	BDL	BDL
Arsenic	As	0.001–0.005	BDL	BDL
Cobalt	Co	0.0002–0.001	BDL	BDL
Mercury	Hg	0.0001–0.0005	BDL	BDL

BDL, below detection Limit; all analyzed metals were below the instrumental detection limits of the ICP-OES method (USEPA Method 200.7, 1994) throughout the study period at both sites.

The concentrations below instrumental detection limits indicate that measurable dissolved contamination by the analyzed metals was limited during the sampling period. Metallurgical, textile, and electroplating industries are commonly associated with elevated metal pollution in aquatic environments [42], 43]. However, the present findings do not exclude the presence of metals below analytical detection limits or their occurrence in suspended particles and sediments. Therefore, the physicochemical variability observed in the present study is interpreted primarily in relation to organic and ionic water-quality characteristics rather than detectable heavy-metal contamination.

Similar findings were reported for the Meghna and Rupsha rivers, where trace metal concentrations remained below permissible limits in areas dominated by household and transport-related discharges [44], 45]. While the results indicate that the Kirtonkhola River currently maintains low heavy metal contamination, continued monitoring is essential because these pollutants can accumulate over time in sediments and aquatic organisms. Heavy metals often exhibit strong particle affinity and may not appear in dissolved form despite being present in suspended matter [42]. Therefore, future studies should include sediment and benthic analyses to provide a more comprehensive understanding of potential long-term accumulation.

Overall, the results suggest that detectable heavy metal contamination was limited during the study period. However, continued monitoring remains important because trace metals may accumulate in sediments and aquatic organisms over time even when dissolved concentrations remain below detection limits.

### 3.6 Correlation among water quality parameters

The correlation analysis among key physicochemical parameters (Table 3) revealed significant relationships that help explain the underlying hydrochemical processes in the Kirtonkhola River system. At Launch Ghat, a nearly perfect positive correlation was found between total dissolved TDS and EC (*r* = 0.995, *p* < 0.001), indicating that ionic concentration is the dominant contributor to conductivity. This strong linear relationship demonstrates that as the total ionic load increases, the water’s ability to conduct electricity also increases, which is consistent with the findings of [46], 47], who observed a similar proportional relationship in tropical rivers.

**Table 3:** Correlation coefficients among various water quality parameters at Launch Ghat and Dopdopia Bridge.

Location	Parameters	EC	TDS	DO	pH
Launch Ghat	EC	1	0.995^b^	−0.065	0.865^b^
	TDS		1	−0.058	0.887^b^
	DO			1	0.069
	pH				1
Dopdopia Bridge	EC	1	0.996^b^	0.117	0.771^b^
	TDS		1	0.105	0.784^b^
	DO			1	0.024
	pH				1

^b^Correlation is significant at the 0.001 level (2-tailed).

pH exhibited a strong positive correlation with EC (*r* = 0.865, *p* < 0.001) and TDS (*r* = 0.887, *p* < 0.001), suggesting that alkaline conditions in the Kirtonkhola River are associated with higher ionic strength and dissolved solids. The positive relationships among pH, EC, and TDS suggest that dissolved ionic constituents played important roles in controlling river water chemistry during the study period. Such behavior is typical in freshwater systems affected by domestic discharge and vessel waste, where alkaline detergents and nutrient-rich effluents increase mineralization and pH [3]. Conversely, DO showed negligible negative correlations with EC (*r* = −0.065) and TDS (*r* = −0.058), indicating little direct association between dissolved ionic concentration and oxygen dynamics at Launch Ghat. The weak inverse relationships between DO and the electrochemical parameters suggest that oxygen dynamics were likely influenced by multiple interacting environmental and hydrological processes, consistent with the results reported by [1], 48]. A weak positive correlation between pH and DO (*r* = 0.069) suggests minor photosynthetic influence, where CO_2_ assimilation by aquatic plants may slightly elevate both pH and oxygen levels [21].

At Dopdopia Bridge, similar but slightly weaker correlations were observed, indicating gradual self-purification downstream. EC and TDS again showed a nearly perfect positive relationship (*r* = 0.996, *p* < 0.001), reinforcing that EC is a robust surrogate for total dissolved ions in the river [49]. Moderate positive correlations were recorded between pH and EC (*r* = 0.771, *p* < 0.001) and between pH and TDS (*r* = 0.784, *p* < 0.001), indicating that the buffering capacity at Dopdopia is still influenced by dissolved carbonate and bicarbonate ions. The positive correlations between pH, EC, and TDS suggest that mineral dissolution and bicarbonate equilibrium govern water chemistry, a finding supported by [2], 5].

In contrast, DO exhibited only a weak positive relationship with EC (*r* = 0.117), TDS (*r* = 0.105), and pH (*r* = 0.024), implying that oxygen dynamics are controlled more by biological and hydrological factors than by ionic composition. Similar weak DO–EC correlations have been reported in polluted rivers where high organic loading increases biological oxygen demand, decoupling oxygen levels from ionic behavior [10], 27]. The weak positive correlations involving DO at Dopdopia Bridge indicate that oxygen dynamics were largely independent of ionic composition and may have been influenced by multiple seasonal, biological, and hydrological processes.

Overall, the hierarchy of correlation strength (TDS ≈ EC > pH > DO) remained consistent across both sampling points, reflecting the strong interdependence among electrochemical parameters, whereas DO appeared to be influenced by additional environmental and hydrological factors beyond dissolved ionic concentration alone. The strong EC–TDS relationship (>0.99) across sites supports the use of EC as a practical indicator of dissolved ion concentration and mineralization in the river system [32]. The positive association between pH and both EC and TDS suggests that dissolved ionic constituents contributed to seasonal variations in river water chemistry during the study period [44].

The weak correlations involving DO indicate that oxygen dynamics were likely influenced by multiple environmental and hydrological processes beyond dissolved ionic concentrations. Previous studies have shown that organic loading, microbial respiration, and reduced re-aeration can influence DO conditions in river systems [7]. In the present study, the generally weak DO relationships and relatively modest differences between Launch Ghat and Dopdopia Bridge suggest that both seasonal hydrological variability and anthropogenic influences may have contributed to the observed oxygen dynamics.

### 3.7 Relationship between TDS and EC

TDS represents the sum of dissolved inorganic and organic substances in water, primarily consisting of carbonates, bicarbonates, chlorides, sulfates, and nitrates of Ca, Mg, Na, and K [50]. It serves as a critical indicator of water quality, affecting both aquatic life and suitability for consumption or irrigation [27]. The relationship between TDS and EC is well established, as EC measures the total ion concentration, which directly influences TDS levels [51].

The scatter plots and regression analysis illustrate a strong positive correlation between EC and TDS at both Launch Ghat (Figure 3A) and Dopdopia Bridge (Figure 3B). The coefficient of determination (*R* ^2^ = 0.983, *p* < 0.001) at both sites indicates that 98.3 % of the variation in TDS can be explained by EC, confirming a highly linear relationship. At Launch Ghat (Figure 3A), TDS values ranged from 80 mg/L to 160 mg/L, while EC varied from approximately 150 μS/cm to 400 μS/cm. Similarly, at Dopdopia Bridge (Figure 3B), TDS values ranged from 80 mg/L to 160 mg/L, with EC fluctuating between 150 μS/cm and 350 μS/cm.

**Figure 3:**
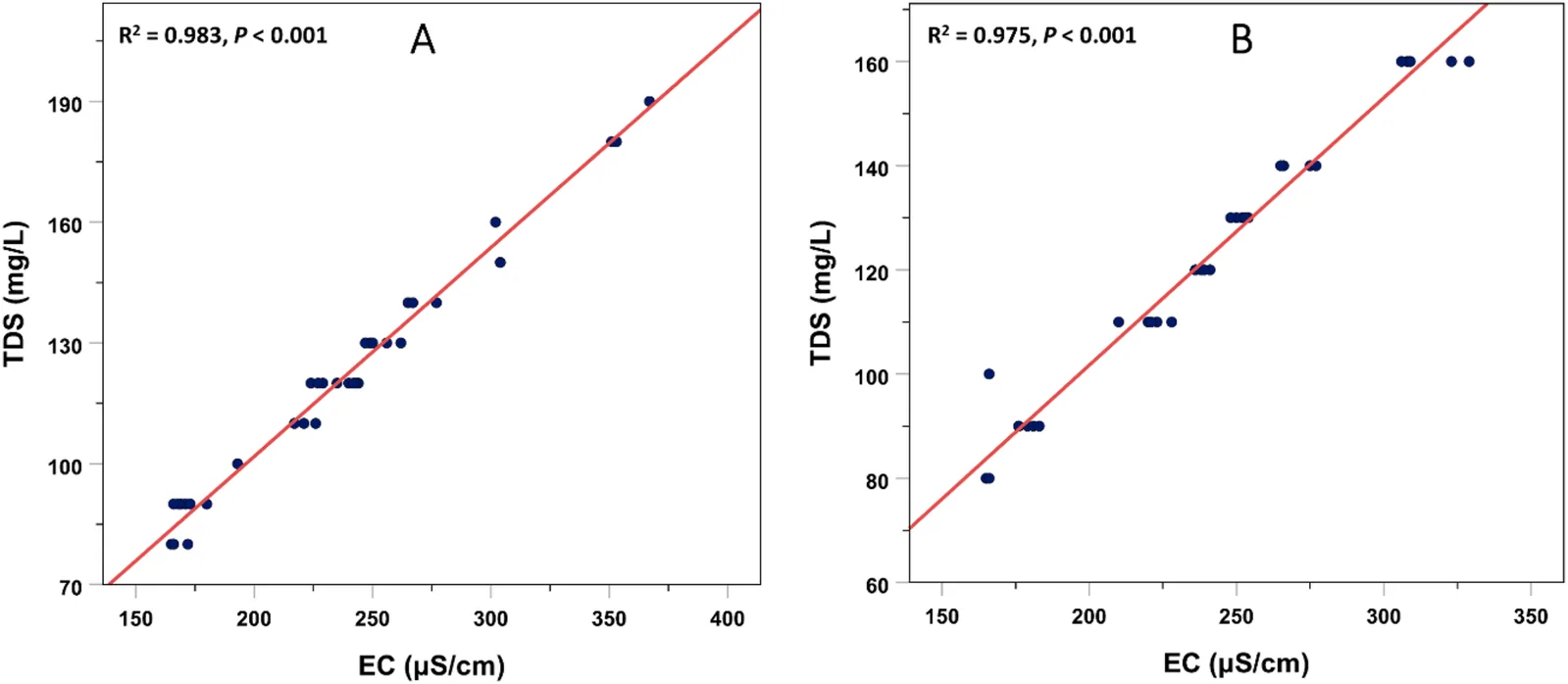
Linear relationship between TDS and EC at Launch Ghat (A) and Dopdopia Bridge (B). Strong positive relationships were observed at both locations (*R* ^2^ = 0.983 and 0.975, respectively; *p* < 0.001).

The strong positive EC–TDS relationship was expected because both parameters reflect the concentration of dissolved ionic constituents. The regression therefore demonstrates consistency between these related physicochemical indicators rather than providing independent evidence of vessel-related pollution. This relationship is consistent with previous studies reporting a strong association between EC and dissolved solids in river systems [47]. The increase in TDS with rising EC suggests that the river’s ionic composition is primarily controlled by dissolved mineral salts rather than suspended particulates or organic matter [52].

Compared to standard water quality guidelines, the measured TDS values were significantly below the threshold levels for drinking water (1,000 mg/L), irrigation (2,000 mg/L), and industrial use (1,500 mg/L) [18], 53]. This indicates a relatively low mineral content in the river, which may limit its buffering capacity but also reduce risks associated with high salinity [54]. Low TDS levels are generally favorable for aquatic life, but some fish species may require a higher ionic concentration for osmoregulation [27].

The seasonal fluctuations observed in TDS levels can be attributed to rainfall, surface runoff, and hydrological dynamics. The lowest TDS values in June correspond to the onset of the monsoon season, when increased freshwater inflow dilutes the river’s dissolved solid concentration. In contrast, the higher TDS values in February and April coincide with the dry season, when reduced river flow and evaporation lead to a higher concentration of dissolved ions [51]. This pattern is consistent with similar river studies worldwide, where TDS and EC exhibit seasonal variability driven by hydrological inputs [49]. The seasonal TDS pattern closely followed EC dynamics, indicating that dissolved ion concentrations were strongly influenced by hydrological variability, particularly rainfall-driven dilution during the monsoon and increased mineral concentration during the dry season.

The implications of low TDS levels extend beyond aquatic health. For irrigation purposes, extremely low TDS levels may indicate poor nutrient content, requiring the addition of fertilizers to enhance soil fertility [55]. However, excessive fertilizer application could contribute to nutrient runoff and water contamination during the monsoon season.

Given the strong relationship between EC and TDS, EC may serve as a practical and rapid indicator of dissolved ionic concentration in future river water monitoring programs [50]. Further studies incorporating long-term monitoring and additional environmental variables would improve understanding of seasonal hydrochemical dynamics and their interactions with other water quality parameters such as DO and pH.

### 3.8 Evaluation of study hypotheses

The findings provided partial support for the study hypotheses. Seasonal variation in EC, TDS, pH, and DO was clearly observed, but differences between the higher-vessel-activity Launch Ghat and the comparatively less impacted Dopdopia Bridge were generally modest. Therefore, the hypothesis that higher vessel activity would consistently correspond to higher EC and TDS and lower DO was only partially supported. The strong EC–TDS relationship reflected the expected association between dissolved ionic constituents, whereas DO showed relatively weak relationships with the ionic parameters. The hypothesis concerning limited heavy-metal contamination was supported insofar as all analyzed metals remained below instrumental detection limits.

### 3.9 Limitations and future directions

This study provides valuable insights into the seasonal dynamics of water quality in the Kirtonkhola River; however, certain limitations should be acknowledged to contextualize the findings and guide future investigations. The sampling design was restricted to two sites (Launch Ghat and Dopdopia Bridge), limiting the spatial coverage needed to fully characterize upstream and downstream pollution gradients. Water sampling was conducted only at the surface level due to a lack of equipment for collecting bottom layer or sediment samples, which could have provided a more complete understanding of pollutant distribution and sediment water interactions (Wetzel [21]). Additionally, volatile organic compounds (VOCs) and other trace contaminants were not analyzed because of limited laboratory facilities. Water temperature and basic hydrological variables, including water depth, flow velocity, and turbulence, were also not measured; therefore, their potential effects on DO, EC, dilution, and seasonal water-quality variability could not be quantitatively distinguished from anthropogenic influences.

Despite these constraints, the study establishes a solid baseline for future research. Long-term and multi-depth monitoring should be undertaken to capture inter-annual and vertical variations in water quality and sediment chemistry [51]. Incorporating heavy metal and organic contaminant analyses would enhance pollution source identification and risk assessment [22]. Future research should also integrate hydrodynamic modeling, Geographic Information System (GIS) based spatial mapping, and machine learning approaches to improve predictive capacity and management of river water quality [47], 49]. Moreover, the inclusion of biological indicators such as microbial diversity, phytoplankton composition, and benthic fauna would provide a holistic perspective on ecosystem health and resilience [27], 36]. Addressing these aspects will contribute to more comprehensive river basin management and support sustainable conservation strategies for the Kirtonkhola River.

## 4 Conclusions

This study evaluated seasonal variations in the water quality of the Kirtonkhola River under contrasting passenger vessel activity conditions at Launch Ghat and Dopdopia Bridge. Seasonal fluctuations in EC, TDS, and DO reflected the combined influence of hydrological variability and anthropogenic inputs. Although Launch Ghat generally exhibited slightly higher EC and TDS values and lower DO concentrations during certain periods, differences between sites were relatively modest, suggesting that seasonal hydrological processes also played important roles in controlling water quality dynamics. The strong EC–TDS relationship indicated that EC may serve as a practical indicator of dissolved ionic concentration in the river system. Heavy metals (Fe, Pb, Zn, As, Co, and Hg) remained below instrumental detection limits throughout the study period. Overall, the findings highlight the importance of continued monitoring and improved waste management practices to support sustainable management of the Kirtonkhola River ecosystem.
